# Event-Related Brain Potentials during a Semantic Priming Task in Children with Learning Disabilities Not Otherwise Specified

**DOI:** 10.1371/journal.pone.0105318

**Published:** 2014-08-21

**Authors:** Thalía Fernández, Juan Silva-Pereyra, Belén Prieto-Corona, Mario Rodríguez-Camacho, Vicenta Reynoso-Alcántara

**Affiliations:** 1 Departamento de Neurobiología Conductual y Cognitiva, Instituto de Neurobiología, Universidad Nacional Autónoma de México, Juriquilla, Querétaro, México; 2 Proyecto de Neurociencias, Facultad de Estudios Superiores (FES) Iztacala, Universidad Nacional Autónoma de México, Estado de México, México; 3 Facultad de Psicología, Universidad Veracruzana, Campus Xalapa, Veracruz, México; University of Western Ontario, Canada

## Abstract

Learning disabilities (LDs) are the most common psychiatric disorders in children. LDs are classified either as “Specific” or “Learning Disorder Not Otherwise Specified”. An important hypothesis suggests a failure in general domain process (i.e., attention) that explains global academic deficiencies. The aim of this study was to evaluate event-related potential (ERP) patterns of LD Not Otherwise Specified children with respect to a control group. Forty-one children (8−10.6 years old) participated and performed a semantic judgment priming task while ERPs were recorded. Twenty-one LD children had significantly lower scores in all academic skills (reading, writing and arithmetic) than twenty controls. Different ERP patterns were observed for each group. Control group showed smaller amplitudes of an anterior P200 for unrelated than related word pairs. This P200 effect was followed by a significant early N400a effect (greater amplitudes for unrelated than related word pairs; 350–550 ms) with a right topographical distribution. By contrast, LD Not Otherwise Specified group did not show a P200 effect or a significant N400a effect. This evidence suggests that LD Not Otherwise Specified children might be deficient in reading, writing and arithmetic domains because of their sluggish shifting of attention to process the incoming information.

## Introduction

### Learning disabilities

Learning disabilities (LDs) are the most common psychiatric disorders in children during their school years [1]. Various groups estimate the prevalence of children with specific learning disabilities to be between 4–10% of all school-aged children [2], [3], [4], but the prevalence of LDs varies widely depending upon operational criteria [5]. According to the American Psychiatric Association [6], LDs are diagnosed when an individual’s achievement on individually administered, standardized tests in reading, mathematics, or written expression is substantially below that expected for their particular age, schooling, and level of intelligence. LDs are classified either as “specific” (reading disorder, math disorder, or disorder of written expression) or “learning disorder not otherwise specified” (when the impairments do not satisfy the criteria of any specific learning disability). This latter category includes observed deficiencies in reading, mathematics, and written expression that may significantly interfere with academic performance even if the individual’s performance on standardized tests is not substantially below the expected performance for the individual’s age, IQ, and grade level.

While efforts have been made to elucidate the underlying cognitive deficits in children with LDs, there is no uniform hypothesis that affords definite knowledge of their causes [7]. Learning disabilities could be due to atypical brain functions, reflected as neurobiological disorders of cognitive processing [8]. There are two main hypotheses with regard to atypical processing patterns underlying LDs [5]. First, the *common deficit hypothesis* postulates that certain patterns of processing are common to all LD children. Second, the *domain-specific cognitive deficit hypothesis* proposes the existence of LD subgroups with specific deficits. Supporting the first hypothesis, Swanson [9] proposed that LD children fail in mechanisms of executive functioning, which also points to working memory (WM) deficits as essential problems in children and adults with LDs [10], [11], specifically in Baddeley’s proposed phonological loop and central executive [5], [12], [13], [14], [15]. Meanwhile, Hari and Renvall [16] postulate sluggish shifting of attention as the source of reading acquisition disorders [17]. Both theoretical frameworks could explain the global deficiencies of LD Not Otherwise Specified.

With respect to the second hypothesis, Siegel [18] contends that there is evidence for independent subgroups of LD children who exhibit distinctive characteristics and existing conditions that consistently predict specific patterns of learning difficulties. For example, children who have reading disabilities have problems with language skills, reading, rapid naming, and spelling. They also have deficiencies in morphological, semantic, and syntactic skills as well as deficits in lexical access, most likely because they have poorer vocabularies [5], [7], [8], [19]. Semantic memory deficiencies arise in this hypothesized subgroup when verbal information is included. Specifically, in LD children and children at risk for dyslexia, some studies that used word pairs or sentences have shown deficiencies in semantic priming tasks [20], [21].

### Semantic priming and ERP

Priming is a phenomenon that, under certain circumstances, can facilitate stimulus processing given the prior processing of a similar stimulus. Although the evaluation of semantic priming frequently employs lexical decision tasks [22], [23], the priming effect can also be evaluated with tasks in which the subject must decide whether two words are semantically related or not. A neurophysiological technique employed to assess different neural processes involved in semantic priming is the method of the Event-Related Potentials (ERPs), which represent brain electrical activity temporally associated with the processing of an event, which can be a sensory, motor, or cognitive process [24]. Among the ERPs studied in Specific LD children are the P200 and N400 components. P200 has been associated with the re-allocation of attentional resources and stimulus evaluation [25]. The P200 amplitude decreases with age [26] but increases with task difficulty [26], [27]. Recent studies have related P200 to an attentional state in preparation for linguistic stimuli that can be anticipated from the sentence context [28], [29]. The N400 ERP component is consistently associated with semantic priming. The N400 is a negative wave that occurs approximately 400 milliseconds after the stimulus in adults [30]. It is elicited during the processing of both written and spoken words. The amplitude of the N400 is modulated as a function of the ease with which a word can be integrated within a higher-order representation of a preceding word or sentence context [31]. Although N400 is sensitive to higher-level factors that have an effect on meaning processing, in some circumstances, it can also be sensitive to lower-level factors (i.e., pre-lexical factors). Typically, the amplitude of the N400 is augmented in response to words that are semantically unprimed (semantically unexpected), i.e., for target words that are not preceded by a related word.

ERP studies analyzing individuals with LDs have also shown contradictory results. Some of these studies showed delayed and attenuated N400 effects during sentence reading [32] and during semantic word priming [27]. A combined functional Magnetic Resonance Image (fMRI) and ERP study revealed reduced N400 effects in dyslexics compared with a control group [33]. In contrast, other studies have found no differences from controls, which could suggest entirely different cognitive profiles in children with LDs. For example, Silva-Pereyra et al. [34] observed normal N400 priming effects in children with reading disorders, and Russeler, Probst, Johannes, and Münte [35] also observed normal N400 effects in adults with reading disorders. Semantic priming seems relatively intact in reading-disabled children; however, neural responses to contextual incongruence are delayed [36].

Surprisingly, ERP studies that include different subtypes of LD children are scarce. Distinct cognitive profiles were observed in LD reading- and arithmetic-disabled children in one ERP semantic priming study [37]. These subgroups were defined by deficient performance on tests of reading and spelling (Group RS) and arithmetic (Group A). Children had to attend to and name pictures and words that varied in their degree of semantic relatedness. In Group RS, children exhibited reduced N400 amplitudes relative to controls, whereas their ERPs in response to pictures were normal, pointing to specific deficiencies in linguistic processing. By contrast, Group A did not exhibit reliable early frontal negative waves, an effect potentially related to a selective attention deficit in these children. These early processing differences were also evidenced by N400 waves of smaller amplitude.

### The present study

Most studies of children with LD have focused on the specific type, especially in those children with reading disorders, which could explain why there is no cognitive or neurobiological profile that describes children with LD Not Otherwise Specified, although they are more prevalent than those with Specific LDs [38]. Previous studies on LD suggest that general deficiencies of children with LD Not Otherwise Specified do involve different cognitive areas related to their school activities (i.e., reading, writing, and arithmetic), because alteration of a general domain process could influence almost every aspect of learning, which would be consistent with the common deficit hypothesis. If results from studies of a Specific LD show deficiencies of semantic processing that are reflected in a decrease of N400 amplitude, it is very probable that children with LD Not Otherwise Specified will also display a pattern of N400 that is different from a control group and probably also from that of children with a specific LD. But more important, if we think that deficiencies of children with LD Not Otherwise Specified are due to a failure in a general domain process or process in common, this fact would be reflected in a different amplitude pattern of the P200, because this ERP component has been associated with the attention process [25]. Therefore, the aim of this study was to assess the ERP pattern of children with LD Not Otherwise Specified during a semantic judgment task.

## Materials and Methods

### Participants

Forty-one children participated in this study. All of the children were volunteers selected from groups of third and fourth graders at two elementary schools. The children had no major cultural disadvantages (in all cases, the mother had at least a primary education, and the family per capita income was above the minimum wage level), all were right-handed, and their neurological exams were normal. All children were assessed with the Child Neuropsychological Assessment (Evaluación Neurológica Infantil, ENI) [39] standardized for the Mexican population, the Wechsler Intelligence Scale for Children – Revised (WISC-R) [40], and the Conners’ Rating Scales – Revised [41]. The children did not show evidence of any psychiatric disorders beyond their LDs, and none met the criteria to be diagnosed with ADHD. Only three domains of the Child Neuropsychological Assessment ENI were evaluated: writing, reading, and arithmetic. Within each domain, we evaluated three variables: accuracy, comprehension, and speed of reading; accuracy, composition and speed of writing as well as counting, numbering (i.e., number comparison), and arithmetic calculations.

Twenty-one children (5 females) with Learning Disabilities Not Otherwise Specified were selected; they had an average age of 9.46±.98 years and an intelligence quotient [40] greater than 80 (Verbal scale 88.29±17.93; Performance scale: 96.29±16.15; Total IQ: 91.52±17.14). These children were referred by a social worker because they had academic performance issues and ranked below the 11^th^ percentile at least on two domains of the Children’s Neuropsychological Evaluation [39].

Twenty right-handed children (11 females) participated in the study as controls (Ctrl). Their ages ranged from 7 to 12 years old (mean 9.18, standard deviation ±1.25), and each of them had a total intelligence quotient that was within the normal range or higher than average (Verbal scale, 107.7±13.67; Performance scale, 106.45±13.25; Total IQ, 107.7±11.95; evaluated with the Wechsler Intelligence Scale for Children-Revised [40]. The children scored within the normal limits in subtests of the Children’s Neuropsychological Evaluation.

**Figure 1:**
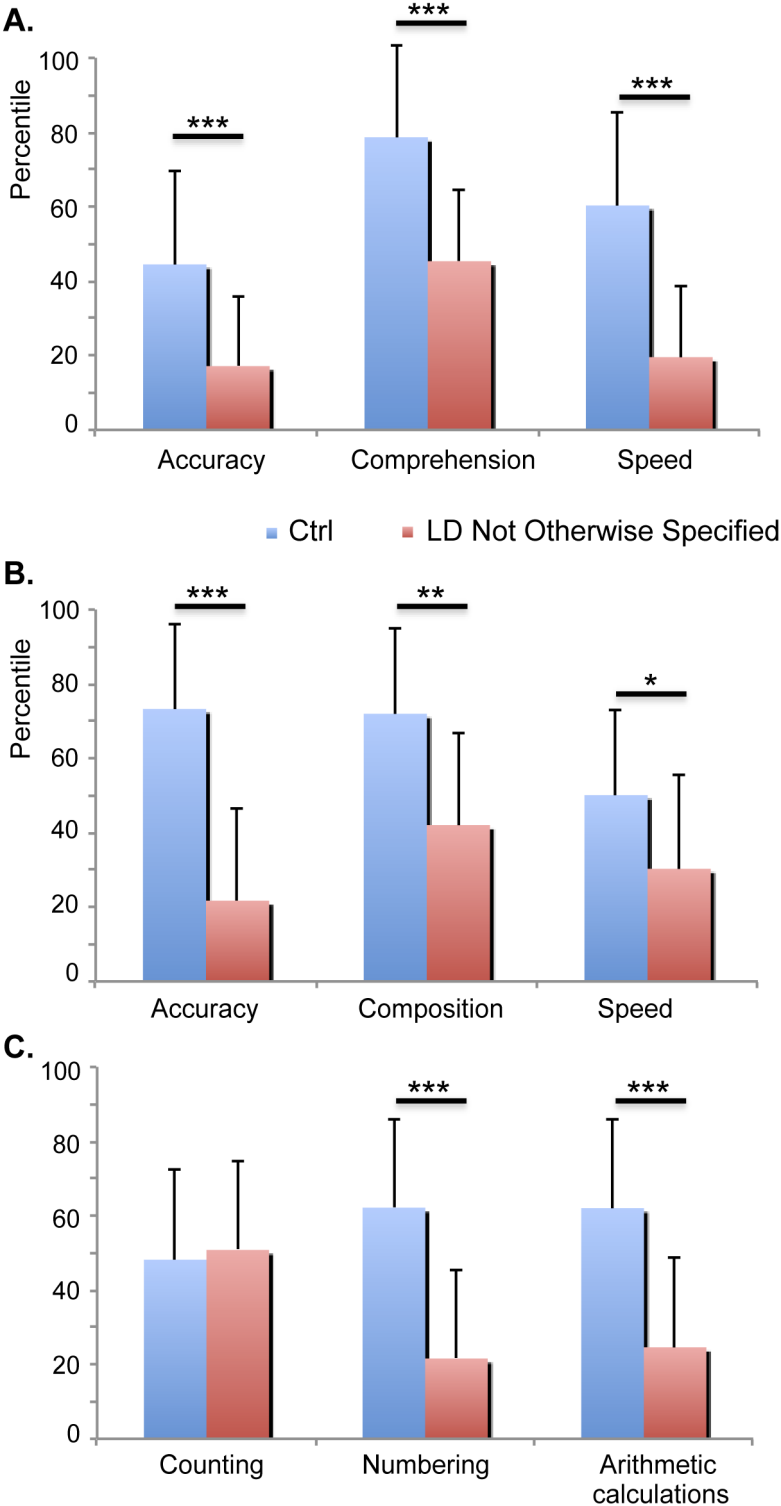
Mean percentile values of groups from subtests of the reading, writing, and arithmetic tests. **A. Reading:** The LD group showed lower scores than Ctrl group in all measurements. **B. Writing:** The LD group mainly showed lower scores for accuracy and composition than the Ctrl group. **C. Arithmetic:** The LD group showed much lower scores on the arithmetic calculations and numbering than and Ctrl group. Significant differences are marked with asterisks: *p<.05, **p<.01, ***p<.001.

Groups did not differ significantly with respect to age (F<1). However, the groups differed in total IQ (F(1,39) = 12.17, p = .001) and if verbal and executive IQ were included as within-subject factor, a Group by IQ subscales interaction was significant (F(1,39) = 4.03, p = .052). The LD Not Otherwise Specified group had lower IQ scores than the Ctrl group (Tukey’s honest significant difference test, MD_HSD_ = 19.41, p<.001 for verbal IQ and MD_HSD_ = 10.16, p = .034 for performance IQ). No child presented with mental retardation. A three-way ANOVA was performed to assess differences between groups across academic skills (i.e., reading, writing, and arithmetic) in the three different measurements of each skill (i.e., accuracy, precision and comprehension-composition for writing-, counting, numbering and arithmetic calculations) and differences are shown in Figure 1.

Significant Group by Academic skills by Measurement interaction (F(4,156) = 7.22, p<.001, epsilon = .989) shows greater scores of Ctrl group than LD for every variable in the reading, writing, and arithmetic domains with the exception of the Counting subtest, where no differences between groups were observed.

### Ethics statement

All the procedures were in line with the Declaration of Helsinki for human research [42]. The Ethics Committee of the Institute of Neurobiology, National Autonomous University of Mexico, approved the experimental protocol. Parents and children provided written informed consent for their participation in this study. Legally, on behalf of children enrolled, parents as their legal guardians signed written informed consent forms.

### Stimuli

A list of 120 pairs of words, including 60 related and 60 unrelated word pairs, were obtained from children’s literature sources [43], [44], [45], [46], [47], [48], [49], [50]. All words had a single meaning (according to the Dictionary of the Royal Spanish Academy, 2003). A word pair was considered related if the words belonged to the same semantic category. Unrelated word pairs did not belong to the same semantic category. Word pairs had to meet the criterion that the second word could not begin or end with the same phoneme as the first. We included several semantic categories: animals, toys, furniture, food, clothing, body parts, musical instruments, professions, places, and tools. All words were singular nouns with one to three syllables, written in Spanish, with no umlauts. Words were displayed in 1-cm uppercase letters in the center of a 14-inch computer monitor (white letters on a black screen). At the viewing distance employed, each letter subtended a visual angle of 0.573×0.573 degrees.

### Procedure

Word pairs were randomly presented. Participants were instructed to respond by pressing one button of a mouse if the second word of the pair was related and a different button if it was not. Because the subjects naturally took the mouse in both hands and used their thumbs to press the buttons, the use of the mouse button was counterbalanced across left- and right-handed subjects.

**Figure 2:**
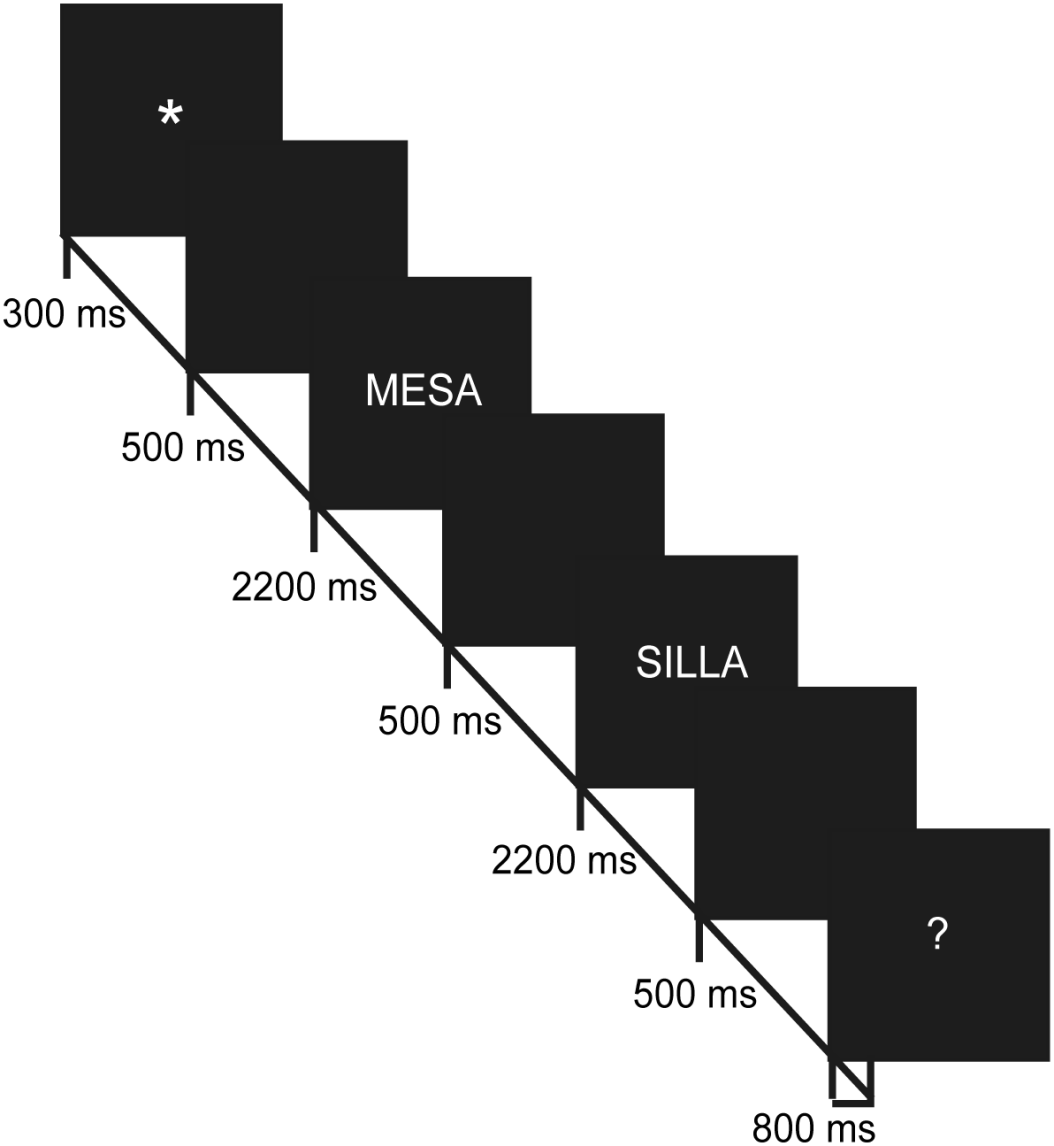
The timing and presentation sequence of stimuli in each trial.

The stimuli were delivered through Mind Tracer software (Neuronic S.A., México D.F., México). Each trial began with the presentation of a warning signal (an asterisk) for 300 ms at the center of a computer monitor. Next, after 500 ms of dark screen, the first word of the pair was presented for 2200 ms; 500 ms later, the second word was presented for 2200 ms. Finally, 500 ms later, a question mark (?) was presented for 800 ms, and an additional 1200 ms was allowed for answering. The children were instructed to respond as rapidly and accurately as possible to each stimulus, but they had to wait to respond until after the question mark appeared. If a child took more than 2 seconds to respond, the trial was considered to be a “no response”, and the presentation of a new sequence was initiated. Figure 2 shows the stimuli presentation sequence.

Before performing the experimental task, each participant was given a short test to verify that he/she understood the task and was familiar with the activity. The subject was comfortably seated in front of the computer monitor at a distance of 50 cm for stimulus presentation. The task was divided into 4 blocks of 30 word pairs each. Each block lasted approximately 4 minutes. A short break was given to the children between blocks. To determine the time stimulus parameters, a pilot study was conducted with 12 adults and then with another sample of 8 elementary school children. From this study, we estimated that the time of presentation of the word needed to be at least 2200 ms to be read by young readers and children with reading disorders.

### ERP recording

EEGs were recorded with a MEDICID-4 system (Neuronic S.A., México D.F., México) from 19 leads of the 10–20 International System (Fp1, Fp2, F3, F4, C3, C4, P3, P4, O1, O2, F7, F8, T3, T4, T5, T6, Fz, Cz, and Pz) in a standard electro-cap (Electro-Cap International Inc., Ohio, USA) referenced to the short-circuited earlobes (A1–A2). The amplifier bandwidth was set between 0.05 and 30 Hz. All electrode impedances were at or below 5 k Ohms, and the signal was amplified with a gain of 20,000. The EEG was digitized at a sampling rate of 200 Hz and stored on a hard disk for further analysis. Blinking and eye movements were monitored from a supra-orbital electrode and from an electrode placed at the external canthus of the right eye. Trials with artifacts due to eye movements or excessive muscle activity were eliminated off-line before averaging. A pre-stimulus time of 100 ms was used to establish the baseline.

Artifact-free EEG segments 1000-ms in length with a 100-ms pre-stimulus time were selected and synchronized with the second word of the pair. At least 25 segments were required from each of the two experimental conditions (i.e., related and unrelated word pairs). Segments were selected only when the answer was correct. Approximately equal numbers of EEG segments were included in the averages for each experimental condition across subjects.

### Data analysis

For behavioral data, the median reaction time (RT) for correct responses was calculated for each subject, and the data were used to perform a two-way ANOVA. The variables included were Group (Ctrl and LD) and Semantic judgment (related and unrelated). The percentages of correct responses were transformed using an ARCSIN [SQRT (percentage/100)] transformation, and these data were used to perform a two-way ANOVA with the same factors used in the RT analysis. Tukey’s honest significant difference post hoc tests were completed after the ANOVA.

**Figure 3:**
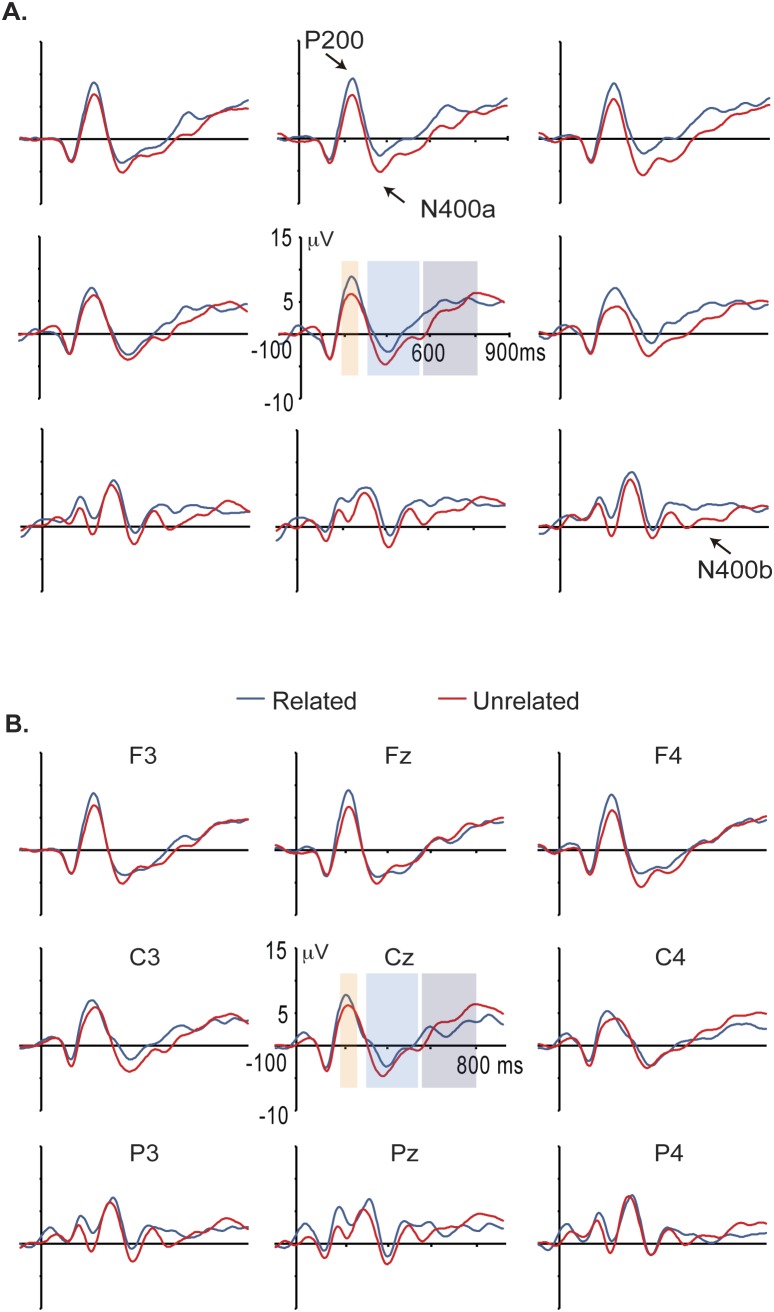
ERP wave grand averages across nine electrode sites of A. Ctrl children and B. LD Not Otherwise Specified children. Responses to related and unrelated word pairs are represented by the blue and red lines respectively. Negativity is plotted downwards. A P200 effect in anterior regions was observed in the Ctrl (i.e., greater amplitudes to related pairs). Unrelated word pairs elicited greater amplitudes of N400a than those elicited by related pairs on anterior right regions in the Ctrl group but this effect was not significant in the LD group.

**Figure 4:**
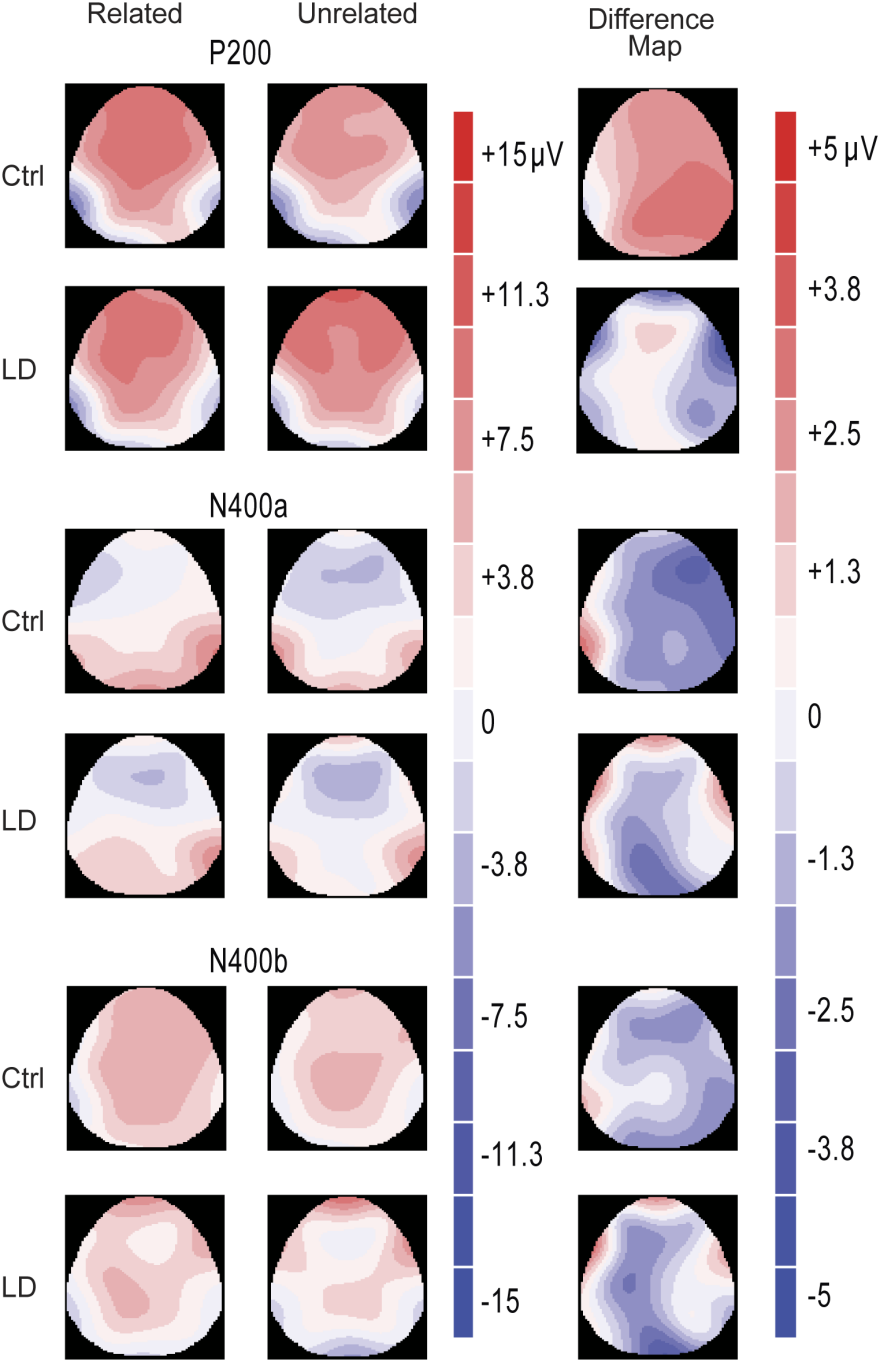
Amplitude Maps per experimental condition and Maps of difference waves for each ERP component in each group. Unrelated word pairs elicited an amplitude effect at approximately 400 ms with right distribution in the Ctrl group.

ERPs from correct responses were obtained for each group (Ctrl and LD) and each experimental condition. Figure 3 shows grand average ERPs and Figure 4 displays the voltage maps of related and unrelated word pairs. Visual inspection reveals that in control group, at approximately 200 milliseconds on frontocentral regions, brainwaves associated with unrelated pairs were smaller (i.e., less positive) than those associated with the related pairs. This effect is commonly referred to as a P200 and this finding is very similar to that reported by Silva-Pereyra et al. [34]. The P200 effect was followed by a typical N400 effect, showing larger amplitudes for unrelated than for related word pairs (i.e., more negative). This effect started at approximately 300-ms and was maintained for more than 500 ms.

According to their appearance in the grand average waveforms, the P200 was considered for analysis as mean amplitude within the interval of 180–250 ms. Due to the long duration of the N400, we decided to divide it into two time windows as others have done [36], thus the N400a was considered the mean amplitude within the interval of 300–550 ms, and the N400b was defined as the mean amplitude within the interval of 555–800 ms.

Separate four-way ANOVAs were performed on amplitude data for each ERP component without midline electrodes using Group as between-subject factor, and Semantic judgment, Hemisphere (left and right) and Electrode site (Fp1, Fp2 F3, F4, C3, C4, P3, P4, F7, F8, T3, T4, T5, T6, O1, O2) as within-subject factors. Three-way ANOVAs were performed on amplitude data for each ERP component with midline electrodes using Group as between-subject factor, and Semantic judgment and Electrode site (Fz, Cz, Pz) as within-subject factors. The Huynh-Feldt epsilon was applied to the degrees of freedom of those analyses with more than one degree of freedom in the numerator. Corrected p-values and epsilon were reported. Tukey’s honest significant difference (HSD) post-hoc tests were completed after the ANOVA.

## Results

### Behavioral data

There was no significant main effect of Group on reaction times (F(1, 39) = 2.91, p = .096) but there was a significant Group by Semantic judgment interaction (F(1, 39) = 4.14, p = .049). Tukey’s HSD post hoc analyses showed priming effects (i.e., faster responses to related than to unrelated pairs) for the LD group (mean of differences: MD_HSD_ = 71.76 ms, p<.001) but not for Ctrl (MD_HSD_ = 26.85 ms, p = .097) group (see Table 1). Reaction time differences between groups were for unrelated pairs (MD_HSD_ = 118.04 ms, p = .05). Transformed percentages of correct responses were employed to perform a two-way ANOVA with the same factors used in the RT analysis. This analysis showed greater percentage of correct responses for the Ctrl group (Mean = 87.33%) than for the LD group (75.11%) (F(1, 39) = 11.78, p = .001), but there was no significant Group by Semantic judgment interaction (F(1, 39) = 1.86, p = .18).

**Table 1:** Behavioral data by Group of children.

		Groups			
		Control		LD Not Otherwise Specified	
		Mean	SD	Mean	SD
RT	Related pairs	522.40	188.50	595.52	167.51
	Unrelated pairs	549.25	192.60	667.29	182.64
% CR	Related pairs	85.74	10.94	74.76	16.72
	Unrelated pairs	88.92	8.47	75.45	13.20

RT = reaction time; % CR = Percentage of Correct responses; SD = standard deviation.

### ERP data

#### P200 (180−250 ms)

In this time window, for the analysis without midline electrodes there was a significant Group by Semantic judgment interaction (F(1, 39) = 5.8, p = .021), which indicated smaller amplitudes of the P200 to Ctrl than LD group for unrelated word-pairs experimental condition (MD_HSD_ = −2.43 µV, p = .03). This interaction also showed smaller amplitudes for unrelated than for related word pairs in the Ctrl group (MD_HSD_ = 1.81 µV, p = .04) but no differences for LD group (MD_HSD_ = −1.08 µV, p = .21). Such P200 effect was lateralized (Group × Semantic judgment × Hemisphere, F(1, 39) = 4.38, p = .043) to the right hemisphere for Ctrl group (MD_HSD_ = 2.3 µV, p = .01). There were no significant Group × Semantic judgment × Electrode site (F<1) and Group × Semantic judgment × Hemisphere × Electrode site interactions (F(7, 273) = 1.22, p = .304, epsilon = .579), and there was no significant main effect of Group (F(1,39) = 1.463, p = .234).

For the ANOVA using midline electrodes, there was no significant main effect of Group (F<1), nor Group by Semantic judgment (F(1, 39) = 2.28, p = .139) and Group by Semantic judgment by Midline electrodes interactions (F<1).

#### N400a (300−550 ms)

For the ANOVA without midline electrodes there was a significant Group by Semantic judgment by Hemisphere interaction (F(1, 39) = 5.41, p = .025). This interaction showed greater amplitudes for unrelated than for related word pairs on the right hemisphere in the Ctrl group (MD_HSD_ = 2.36 µV, p = .036) but no differences for LD group (MD_HSD_ = .235 µV, p = .83). This ANOVA also revealed no other significant interactions (Group × Semantic judgment interaction F<1; Group × Semantic judgment × Electrode site interaction F(7, 273) = 1.12, p = .341, epsilon = .372; Group × Semantic judgment × Hemisphere × Electrode site interaction F(7, 273) = 1.733, p = .127, epsilon = .733). No main effect of Group (F<1) was observed.

For the analysis using midline electrodes, there was no significant main effect of Group (F<1), and Group by Semantic judgment (F<1) and Group by Semantic judgment by Midline electrodes interactions (F(2, 78) = 1.837, p = .171, epsilon = .88).

#### N400 b (555−800 ms)

In this time window, there was no significant main effect of Group (F<1) and there were no significant interactions (Group × Semantic judgment interaction F<1; Group × Semantic judgment × Electrode site F<1; Group × Semantic judgment × Hemisphere F(1,3 9) = 1.722, p = .197; Group × Semantic judgment × Hemisphere × Electrode site interaction F<1).

For the analysis using midline electrodes there was a marginal main effect of Group (F(1, 39) = 3.58, p = .066), but no significant interactions (Group × Semantic judgment F<1; Group × Semantic judgment × Midline electrodes F<1).

### Data reanalysis separating LD Not Otherwise Specified into two groups

**Figure 5:**
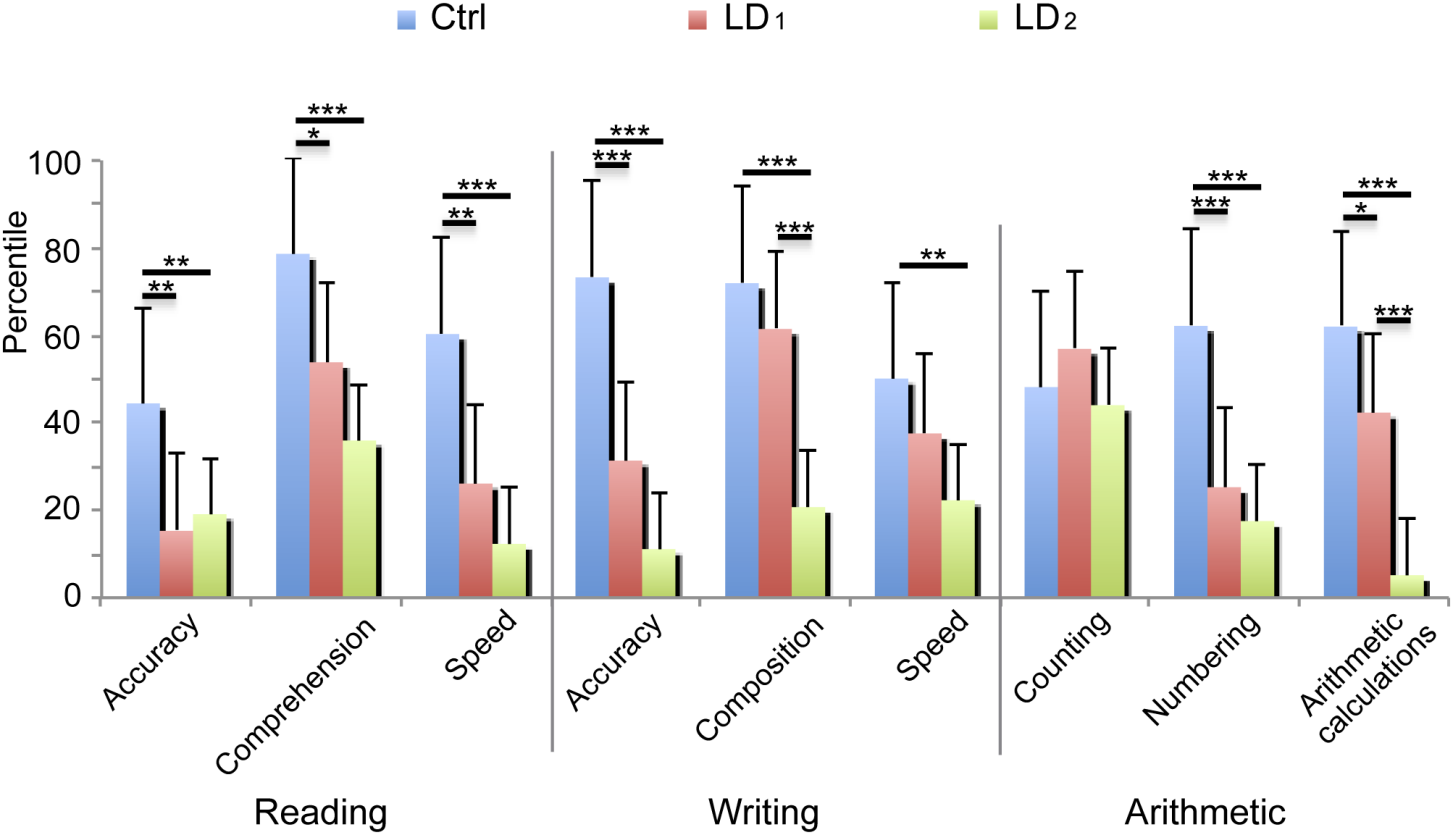
Mean percentile values of three groups (Ctrl, LD_1_ and LD_2_) from all subtests of the reading, writing, and arithmetic tests. LD_1_ group shows greater scores than LD_2_ group only in Composition subtest from Writing and Arithmetic calculations subtest from Arithmetic. Significant differences are marked with asterisks: *p<.05, **p<.01, ***p<.001.

A hierarchical cluster analysis was applied to identify possible homogeneous subgroups of children with LD Not Otherwise Specified. Percentiles from three tests of the neuropsychological battery ENI (reading comprehension, writing composition, and arithmetic calculations) were used in this analysis, which was completed using the Ward method with a measure of squared Euclidean distance. Once the clusters were obtained, a one-way ANOVA was performed to assess differences between groups in academic skills (reading, writing, and arithmetic) as shown in Figure 5. The Huynh-Feldt epsilon was applied to the degrees of freedom of those analyses with more than one degree of freedom in the numerator and it was reported. Tukey’s honest significant difference (HSD) post-hoc tests were completed after the ANOVA.

A visual inspection of the dendrogram revealed two independent clusters almost equal in size and with different characteristics. The following two groups were obtained: LD_1_: n = 11 (4 female, age 9.36±.77; total IQ: 99.36±10.41; verbal IQ: 95.36±18.53; performance IQ: 103.82±17.53); and LD_2_: n = 10 (1 female, age 9.57±1.2; total IQ: 82.9±10.89; verbal IQ: 80.5±14.31; performance IQ: 88±9.63). As it can be seen at Figure 5, there were differences between LD subgroups in several subscales of the neuropsychological test (F(8, 152) = 4.64, p<.001, epsilon = .975). LD_2_ group showed lower scores in writing composition, and arithmetic calculations than LD_1_ who showed lower scores in reading accuracy, reading speed, reading comprehension, writing accuracy, numbering and arithmetic calculations than the Ctrl group. The LD_2_ group showed lower scores in reading accuracy, reading comprehension, reading speed, writing accuracy, writing composition, writing speed, numbering, and arithmetic calculations than the Ctrl group.

Groups (Ctrl, LD_1_ LD_2_) did not differ significantly with respect to age (F<1), however, the groups differed in total IQ (F(2, 38) = 10.86, p<.001), Ctrl had greater IQ scores than LD_2_ (MD_HSD_ = 24.8, p<.001), LD_1_ also had significantly greater IQ scores than LD_2_ (MD_HSD_ = 16.464, p = .009), but there were no differences between Ctrl and LD_1_ (MD_HSD_ = 8.336, p = .114). ANOVA results including verbal and performance IQ scores as within-subject factor showed also differences between groups (F(2, 38) = 11.156, p<.001) as the previous result only using total IQ scores as variable. There was no Group by Verbal and Performance IQ scores interaction (F(2, 38) = 1.98, p = .153).

### Behavioral data

A two-way ANOVA was performed with the behavioral data using the two groups obtained from cluster analysis and our Ctrl group (Ctrl, LD_1_, and LD_2_) as between-subject factor and Semantic judgment (related and unrelated) as within-subject factor. There were no significant main effect of Group on reaction times (F(2, 38) = 1.525, p = .23) neither significant Group by Semantic judgment interaction (F(2, 38) = 2.06, p = .14). However, there was a significant main effect of Group regarding percentage of correct responses (F(2, 38) = 8.34, p = .001). This result indicates a greater percentage of correct responses for the Ctrl group relative to the LD_2_ group (MD_HSD_ = .24, p<.001), LD_1_ also displayed greater percentage than LD_2_ group (MD_HSD_ = .134, p = .05), but there were no significant differences between Ctrl and LD_1_ (MD_HSD_ = .11, p = .071). The Group by Semantic judgment interaction was not significant for the percentage of correct responses (F<1).

### ERP data

Three time windows were used as in the previous analysis (i.e., P2: 180–250 ms, N400a: 300–550 ms, and N400b: 555–800 ms) and Group factor now included: Ctrl, LD_1_ and LD_2_.

### P200

ANOVA without midline electrodes showed a significant Group by Semantic judgment interaction (F(2,38) = 6.64, p = .003), showing smaller amplitudes of the P200 to Ctrl than LD_2_ group for unrelated word-pairs experimental condition (MD_HSD_ = −3.59 µV, p = .008). This interaction also shows smaller amplitudes for unrelated than for related word pairs in the Ctrl group (MD_HSD_ = 1.81 µV, p = .03), in contrast, LD_2_ group displayed the inverse pattern (MD_HSD_ = −3.19 µV, p = .008) and LD_1_ showed no differences between conditions (MD_HSD_ = .85 µV, p = .44). Results also showed no significant Group by Semantic judgment by Electrode site (F<1), Group by Semantic judgment by Hemisphere (F(2, 38) = 2.14, p = .13) and Group by Semantic judgment by Hemisphere by Electrode site (F(14, 266) = 1.19, p = .308, epsilon = .567) interactions, and there was no significant main effect of Group (F<1).

For the analysis using midline electrodes there was no significant main effect of Group (F<1), nor Group by Semantic judgment (F(2, 38) = 2.12, p = .135) and Group by Semantic judgment by Midline electrodes interactions (F<1).

### N400a

Analysis without including midline electrodes showed that Group by Semantic judgment by Hemisphere interaction was marginally significant (F(2, 38) = 2.9, p = .067). This interaction shows greater amplitudes for LD_1_ than LD_2_ in unrelated word pairs on the left hemisphere (MD_HSD_ = −3.203 µV, p = .045). This effect also shows greater amplitudes for unrelated than related word pairs for Ctrl group on the right hemisphere (MD_HSD_ = 2.36 µV, p = .033) in contrast to LD_1_ (MD_HSD_ = 1.72 µV, p = .24) and LD_2_ (MD_HSD_ = −1.40 µV, p = .36) where no differences were observed. This ANOVA also revealed no other significant interactions (Group × Semantic judgment interaction F(2, 38) = 1.95, p = .16; Group × Semantic judgment × Electrode site interaction F(14, 266) = 1.11, p = .363, epsilon = .382; Group × Semantic judgment × Hemisphere × Electrode site interaction F(14, 266) = 1.35, p = .203, epsilon = .74). No main effect of Group (F<1) was observed.

For the analysis using midline electrodes there was no significant main effect of Group (F<1), nor Group by Semantic judgment (F<1) and Group by Semantic judgment by Midline electrodes (F(4, 76) = 1.88, p = .131, epsilon = .89).

### N400b

There was no significant main effect of Group (F<1) and there were no significant interactions for ANOVA without midline electrodes (Group × Semantic judgment interaction F<1; Group × Semantic judgment × Electrode site interaction F<1; Group × Semantic judgment × Hemisphere interaction F<1; Group × Semantic judgment × Hemisphere × Electrode site interaction F(14, 266) = 1.36, p = .22, epsilon = .566).

For the analysis using midline electrodes there was no significant main effect of Group (F(2, 38) = 1.93, p = .16) nor significant interactions (Group × Semantic judgment F<1; Group × Semantic judgment × Midline electrodes F<1).

## Discussion

The present study aimed to compare the ERP pattern of children with LD Not Otherwise Specified to that of a control group during a semantic judgment task, because there are no studies considering this type of LD. We think that general deficiencies across cognitive areas in LD Not Otherwise Specified are due to a general domain process failure, so first we expected children with LD and controls would show different P200 pattern and second, this attention problem (i.e., without any evidence of Attention Deficit Disorder) would probably also be reflected in a different N400 pattern, all of this in way similar to findings in Specific LD. Our results support this idea. LD Not Otherwise Specified children showed no differences in the P200 between related and unrelated word pairs. By contrast Ctrl group displayed larger P200 amplitudes in response to related than to unrelated word pairs, which was mainly observed over the frontal regions, as found previously in normal readers [34]. This group difference at P200 has been found in other studies using children with Specific LD i.e., reading disabled children [37]. Enhanced P200 responses have often been observed for words in constraining sentence contexts, perhaps reflecting a preparatory attentional response elicited by language contexts that generate a strong expectation for particular upcoming stimuli [28], [29], [51]. The formation of strong context-based expectations for upcoming words seems to change how the perceptual processing system allocates attention and analyzes subsequent stimuli [52]. Thus, a P200 amplitude pattern in children with LD Not Otherwise Specified or children with Specific LD that differs from the Ctrl group could reveal an attention deficit, i.e., a deficit in a general domain process. In fact, a previous study showed reduced P200 activation of the right superior parietal region (BA7) in poor readers relative to normal readers in cue condition trials during a visual continuous performance task [53]. Such P200 amplitude pattern in LD children with reading disabilities could support the common deficit hypothesis. So, differences in P200 found between groups suggest important attention differences when children include a word in a semantic category (as Federmeier’s studies have indicated).

Probably these P200 ERP differences are reflected in important cognitive profile differences between groups; whereas the LD Not Otherwise Specified group showed severe deficiencies in all areas evaluated (i.e., reading speed and comprehension, writing accuracy and composition, numbering and arithmetic), the Ctrl group showed normal scores, and in fact, reflects differences at other ERP components related to later cognitive processes such as lexical and semantic processes. Thus, this study shows that Ctrl group displayed N400 effects (i.e., larger amplitudes for unrelated than related pairs) at 300 to 550 ms in contrast to our LD Not Otherwise Specified children who displayed no significant effect, as many others have shown with semantic priming tasks [34], [36] but in children with Specific LD. At 555 to 800 ms however, there were no differences between groups. Here, N400 topographic differences between the groups were shown at plots of ERP grand averages where the Ctrl group showed an N400 effect on frontal sites, in line with previous studies performed with normal subjects [34], [36]; however significant statistical differences were only observed on the right hemisphere.

Our findings certainly support the common deficit hypothesis, which is compatible with the Working Memory deficits [9] and Sluggish Attentional Shifting (SAS) [16] frameworks because it clusters all Specific LDs, and we would include here children with LD Not Otherwise Specified, into a common cognitive impairment. For example, according to SAS, when LD Not Otherwise Specified children deal with the stimulus sequences or word pairs, first, they have to efficiently read each stimulus presented, and after that, children have to judge if the pair belongs to the same semantic category, so their automatic attention system cannot disengage fast enough from one item to the next one, yielding slow and degraded processing. SAS is assumed to distort cortical networks such as those that support sublexical auditory-phonological and visual-orthographic representations. Consequently, it is possible to suggest that the global deficits in LD Not Otherwise Specified can be linked to a generally inefficient multi-sensory processing of perceptual stimulus. However, our findings cannot discard the idea that there is a failure in mechanisms of executive functioning of working memory in children with LD Not Otherwise Specified [9], but it would be necessary to design an experiment ad hoc to link children’s deficiencies to working memory mechanisms.

The altered attention pattern in children with LD Not Otherwise Specified may be due to multiple factors such as a great heterogeneity in their brain maturation. In fact, it has been suggested that the neurobiological maturation of cognition is reflected by the long time brain specialization areas take to mature [54]. The last brain area to mature is the frontal lobe, which is the region where one component of the P200 has its source [53]. Thus, these lower scores in the LD Not Otherwise Specified group probably reflect a lag in prefrontal maturation [55]. In fact, a large percentage of children with LD show EEG-delayed maturation [56], [57], characterized by an excess of theta activity in frontal regions [58].

Now, small differences in the amplitude in the window of N400 found between groups may be due to other factors that arise as a consequence of an alteration in the attention process. That is, an initial deficiency in the attention mechanism may broadly directly influence other processes, such as lexical access and semantic judgment required to execute the task. Although statistically significant differences have not been shown between groups and between experimental conditions regarding behavioral results, in general children with LD Not Otherwise Specified show a lower number of correct answers. One explanation that would account for the bare differences in the N400 effect in this study and even others [35], [36], [40] whose results are not consistent [37], [39], may be that the greater amplitude of P200 to unrelated stimuli makes it remain above the baseline that would be the beginning of N400. Therefore, by comparing both conditions (related vs. non-related), differences in amplitude would be reduced between the conditions of this latter component. One possibility that may be added to the above would be that the IQ scores may be an important marker of disadvantage of the LD Not Otherwise Specified, since statistically significant differences have been observed in scores in the verbal scale of IQ between groups of children. More so, upon dividing the LD into two groups according to the neuropsychological profile of ENI, group LD_2_ that had lower IQ scores and more severe cognitive deficit presented an inverse pattern of P200 (i.e., greater amplitude for unrelated pairs than related ones) regarding controls, a fact that may influence in the N400 effect, as mentioned above, since LD_1_ displayed a greater amplitude than LD_2_ for unrelated pairs. These results suggest that, regardless of whether the LD is a type not otherwise specified or specific, the degree of deterioration in children’s skills becomes most important, since it is clear that LD_2_ group elicits a worst brain response than LD_1_ in this semantic priming task.

In summary, children with LD Not Otherwise Specified showed an altered P200 that is similar to that reported in children with Specific LD (i.e., reading disabled). It is probable that the alteration in the N400 effect in these children is due to the lack of attention that is previous to semantic judgment required in the priming task. It is also feasible that differences in the N400 effect are due to a wave overlapping with the previous P200, a heterogeneity in the maturation of the frontal lobe, or even a low IQ.

## Conclusions

According to the definition provided by the DSM-IV [6], children with LD evaluated in this study can be characterized as “Not Otherwise Specified”. This kind of LD probably shows an important deficit in preparatory attention provoked by context and generating a strong expectation for the stimuli that are to appear. Similar to that reported for Specific LD, lack of attention in children with LD Not Otherwise Specified may be common to all LDs and affect, in a snowball effect, other cognitive processes such as lexical access and, later, semantic judgment. It will be necessary to obtain ERPs during writing and arithmetic tasks to further test the hypothesis of common deficit.

## References

[1] Handler SM, Fierson WM (2011) Learning disabilities, dyslexia, and vision. Pediatrics 127: e818–856. 21357342 10.1542/peds.2010-3670

[2] Fletcher TV, Kaufman de Lopez CK (1995) A Mexican perspective on learning disabilities. J Learn Disabil 28: 530–534, 544. 8530894 10.1177/002221949502800901

[3] Lagae L (2008) Learning disabilities: definitions, epidemiology, diagnosis, and intervention strategies. Pediatr Clin North Am 55: 1259–1268, vii. 19041456 10.1016/j.pcl.2008.08.001

[4] Sotelo-Dynega M, Flanagan D, Alfonso V (2010) Overview Of Specific Learning Disabilities. In: Flanagan D, Alfonso V, editors. Essentials of Specific Learning Disability Identification. Hoboken, New Jersey: John Wiley & Sons, Inc. 1–20.

[5] Landerl K, Fussenegger B, Moll K, Willburger E (2009) Dyslexia and dyscalculia: two learning disorders with different cognitive profiles. J Exp Child Psychol 103: 309–324. 19398112 10.1016/j.jecp.2009.03.006

[6] American Psychiatric Association (2002) Manual Diagnóstico y Estadístico de los trastornos mentales, DSM-IV-TR. Barcelona: Masson.

[7] Büttner G, Hasselhorn M (2011) Learning Disabilities: Debates on definitions, causes, subtypes, and responses. International Journal of Disability, Development and Education 58: 75–87.

[8] Silver CH, Ruff RM, Iverson GL, Barth JT, Broshek DK, et al (2008) Learning disabilities: the need for neuropsychological evaluation. Arch Clin Neuropsychol 23: 217–219. 17977692 10.1016/j.acn.2007.09.006

[9] Swanson HL (1987) Information processing theory and learning disabilities: a commentary and future perspective. J Learn Disabil 20: 155–166. 3549949 10.1177/002221948702000303

[10] Berninger V (2008) Defining and differentiating dysgraphia, dyslexia, and language learning disability within a working memory model. In: Mody M, Silliman E, editors. Brain, behavior, and learning in language and reading disorders. New York: The Guilford Press. 103–134.

[11] Swanson HL, Siegel L (2001) Learning disabilities as a working memory deficit. Issues Educ Contrib Educ Psychol: 1–48.

[12] Fletcher J (1985) Memory for verbal and nonverbal stimuli in learning disability subgroups: analysis by selective reminding. J Exp Child Psychol 40: 244–259. 4045379 10.1016/0022-0965(85)90088-8

[13] Maehler C, Schuchardt K (2011) Working Memory in Children with Learning Disabilities: Rethinking the criterion of discrepancy. International Journal of Disability, Development and Education 58: 5–17.

[14] Swanson HL (2012) Cognitive profile of adolescents with math disabilities: are the profiles different from those with reading disabilities? Child Neuropsychol 18: 125–143. 21967554 10.1080/09297049.2011.589377

[15] Swanson HL, Stomel D (2012) Learning Disabilities and Memory. In: Wong B, Butler D, editors. Learning about learning disabilities. USA: Elservier.

[16] Hari R, Renvall H (2001) Impaired processing of rapid stimulus sequences in dyslexia. Trends Cogn Sci 5: 525–532. 11728910 10.1016/s1364-6613(00)01801-5

[17] Lallier M, Tainturier MJ, Dering B, Donnadieu S, Valdois S, et al (2010) Behavioral and ERP evidence for amodal sluggish attentional shifting in developmental dyslexia. Neuropsychologia 48: 4125–4135. 20933526 10.1016/j.neuropsychologia.2010.09.027

[18] Siegel L (2003) Learning Disabilities. In: Reynolds WM, Miller, G.E, Weiner, I.B., editor. Handbook of Psychology, Educational Psychology. Hoboken, New Jersey: John Wiley & Sons, Inc. 455–486.

[19] Shafrir U, Siegel LS (1994) Subtypes of learning disabilities in adolescents and adults. J Learn Disabil 27: 123–134. 8195688 10.1177/002221949402700207

[20] Rodríguez M, Prieto B, Bernal J, Marosi E, Yáñez G, et al. (2006) Language Event-Related Potentials in Poor Readers. In: Randall SV, editor. Learning disabilities New research. New York, USA: Nova Science, Publishers, Inc. 187–217.

[21] Torkildsen JvK, Syversen G, Simonsen HG, Moen I, Lindgren M (2007) Electrophysiological correlates of auditory semantic priming in 24-month-olds. Journal of Neurolinguistics 20: 332–351.

[22] Meyer DE, Schvaneveldt RW (1971) Facilitation in recognizing pairs of words: evidence of a dependence between retrieval operations. J Exp Psychol 90: 227–234. 5134329 10.1037/h0031564

[23] Neely J (1991) Semantic priming effects in visual Word recognition: a selective review of current findings and theories; Besner D, Humphreys G, editors. Hillsdale, New Jersey: Lawrence Erlbaum Associates.

[24] Picton TW, Bentin S, Berg P, Donchin E, Hillyard SA, et al (2000) Guidelines for using human event-related potentials to study cognition: recording standards and publication criteria. Psychophysiology 37: 127–152. 10731765

[25] Johnson R Jr (1989) Developmental evidence for modality-dependent P300 generators: a normative study. Psychophysiology 26: 651–667. 2629013 10.1111/j.1469-8986.1989.tb03167.x

[26] Taylor MJ, Khan SC (2000) Top-down modulation of early selective attention processes in children. Int J Psychophysiol 37: 135–147. 10832000 10.1016/s0167-8760(00)00084-2

[27] Stelmack RM, Saxe BJ, Noldy-Cullum N, Campbell KB, Armitage R (1988) Recognition memory for words and event-related potentials: a comparison of normal and disabled readers. J Clin Exp Neuropsychol 10: 185–200. 3350919 10.1080/01688638808408235

[28] Federmeier KD, Mai H, Kutas M (2005) Both sides get the point: hemispheric sensitivities to sentential constraint. Mem Cognit 33: 871–886. 10.3758/bf03193082 16383175

[29] Wlotko EW, Federmeier KD (2007) Finding the right word: hemispheric asymmetries in the use of sentence context information. Neuropsychologia 45: 3001–3014. 17659309 10.1016/j.neuropsychologia.2007.05.013 PMC2066191

[30] Kutas M, Federmeier KD (2011) Thirty years and counting: finding meaning in the N400 component of the event-related brain potential (ERP). Annu Rev Psychol 62: 621–647. 20809790 10.1146/annurev.psych.093008.131123 PMC4052444

[31] Kutas M, Federmeier KD (2000) Electrophysiology reveals semantic memory use in language comprehension. Trends Cogn Sci 4: 463–470. 11115760 10.1016/s1364-6613(00)01560-6

[32] Brandeis D, Vitacco D, Steinhausen HC (1994) Mapping brain electric micro-states in dyslexic children during reading. Acta Paedopsychiatr 56: 239–247. 8079644

[33] Schulz E, Maurer U, van der Mark S, Bucher K, Brem S, et al (2008) Impaired semantic processing during sentence reading in children with dyslexia: combined fMRI and ERP evidence. Neuroimage 41: 153–168. 18378166 10.1016/j.neuroimage.2008.02.012

[34] Silva-Pereyra J, Rivera-Gaxiola M, Fernandez T, Diaz-Comas L, Harmony T, et al (2003) Are poor readers semantically challenged? An event-related brain potential assessment. Int J Psychophysiol 49: 187–199. 14507438 10.1016/s0167-8760(03)00116-8

[35] Russeler J, Probst S, Johannes S, Munte T (2003) Recognition memory for high- and low-frequency words in adult normal and dyslexic readers: an event-related brain potential study. J Clin Exp Neuropsychol 25: 815–829. 13680459 10.1076/jcen.25.6.815.16469

[36] Jednorog K, Marchewka A, Tacikowski P, Grabowska A (2010) Implicit phonological and semantic processing in children with developmental dyslexia: evidence from event-related potentials. Neuropsychologia 48: 2447–2457. 20430041 10.1016/j.neuropsychologia.2010.04.017

[37] Greenham SL, Stelmack RM, van der Vlugt H (2003) Learning disability subtypes and the role of attention during the naming of pictures and words: an event-related potential analysis. Dev Neuropsychol 23: 339–358. 12740189 10.1207/S15326942DN2303_2

[38] Dirks E, Spyer G, van Lieshout EC, de Sonneville L (2008) Prevalence of combined reading and arithmetic disabilities. J Learn Disabil 41: 460–473. 18768777 10.1177/0022219408321128

[39] Matute E, Rosselli M, Ardila A, Ostrosky-Solís F (2008) Evaluación Neuropsicológica Infantil (ENI). México D.F: Manual Moderno.

[40] Weschler D (2001) Escala de inteligencia de Weschler para niños-revisada (WISC-R). México D.F: Manual Moderno.

[41] Conners K (1997) Conners’ rating Scales-Revised. Technical Manual. New York: Multi-health system. Inc.

[42] World Medical Association (2004) Declaration of Helsinki: ethical principles for medical research involving human subjects. J Int Bioethique 15: 124–129. 15835069

[43] Ahumada R, Montenegro A (1990) Juguemos a leer: libro de lectura y manual de ejercicios. México, D.F: Trillas.

[44] Ahumada R, Montenegro A (2007) Juguemos a leer: libro de lectura y manual de ejercicios. México, D.F: Trillas.

[45] Mondada A (1992) Prácticas de ortografía, 3.Ortografía funcional para el tercer grado de enseñanza primaria con base en cuadros ortográficos. México D.F: Fernández Editores.

[46] Mondada A (1992) Prácticas de ortografía, 2.Ortografía funcional para el segundo grado de enseñanza primaria con base en cuadros ortográficos. México, D.F: Fernández Editores.

[47] Mondada A (1992) Prácticas de ortografía, 4.Ortografía funcional para el cuarto grado de enseñanza primaria con base en cuadros ortográficos. México, D.F: Fernández Editores.

[48] Mondada A (1992) Prácticas de ortografía, 5.Ortografía funcional para el quinto grado de enseñanza primaria con base en cuadros ortográficos. México, D.F: Fernández Editores.

[49] Mondada A (1992) Prácticas de ortografía, 6.Ortografía funcional para el sexto grado de enseñanza primaria con base en cuadros ortográficos. México, D.F: Fernández Editores.

[50] Pestum J (1996) Maya y el truco para hacer la tarea. México D.F: Fondo de Cultura Económica.

[51] Federmeier KD, Kutas M (2002) Picture the difference: electrophysiological investigations of picture processing in the two cerebral hemispheres. Neuropsychologia 40: 730–747. 11900725 10.1016/s0028-3932(01)00193-2

[52] Huang HW, Lee CL, Federmeier KD (2010) Imagine that! ERPs provide evidence for distinct hemispheric contributions to the processing of concrete and abstract concepts. Neuroimage 49: 1116–1123. 19631274 10.1016/j.neuroimage.2009.07.031 PMC2782386

[53] Silva-Pereyra J, Bernal J, Rodriguez-Camacho M, Yanez G, Prieto-Corona B, et al (2010) Poor reading skills may involve a failure to focus attention. Neuroreport 21: 34–38. 19996811 10.1097/WNR.0b013e328332c566

[54] Silva-Pereyra J, Rivera-Gaxiola M, Kuhl PK (2005) An event-related brain potential study of sentence comprehension in preschoolers: semantic and morphosyntactic processing. Brain Res Cogn Brain Res 23: 247–258. 15820632 10.1016/j.cogbrainres.2004.10.015

[55] Segalowitz SJ, Wagner WJ, Menna R (1992) Lateral versus frontal ERP predictors of reading skill. Brain Cogn 20: 85–103. 1389125 10.1016/0278-2626(92)90063-r

[56] Harmony T, Marosi E, Diaz de Leon AE, Becker J, Fernandez T (1990) Effect of sex, psychosocial disadvantages and biological risk factors on EEG maturation. Electroencephalogr Clin Neurophysiol 75: 482–491. 1693893 10.1016/0013-4694(90)90135-7

[57] John ER, Prichep L, Ahn H, Easton P, Fridman J, et al (1983) Neurometric evaluation of cognitive dysfunctions and neurological disorders in children. Prog Neurobiol 21: 239–290. 6665163 10.1016/0301-0082(83)90014-x

[58] Fernandez T, Harmony T, Fernandez-Bouzas A, Silva J, Herrera W, et al (2002) Sources of EEG activity in learning disabled children. Clin Electroencephalogr 33: 160–164. 12449846 10.1177/155005940203300405

